# A Real-Time Mismatch Detection Method for Underwater Database-Referenced Navigation

**DOI:** 10.3390/s19020307

**Published:** 2019-01-14

**Authors:** Tian Dai, Lingjuan Miao, Yanbing Guo

**Affiliations:** School of Automation, Beijing Institute of Technology, Beijing 100081, China; miaolingjuan@bit.edu.cn (L.M.); 20080089@bit.edu.cn (Y.G.)

**Keywords:** database-referenced navigation, mismatch detection, underwater navigation, inertial navigation system, autonomous underwater vehicles

## Abstract

Database-referenced navigation (DBRN) using geophysical information is often implemented on autonomous underwater vehicles (AUVs) to correct the positional errors of the inertial navigation system (INS). The matching algorithm is a pivotal technique in DBRN. However, it is impossible to completely eliminate mismatches in practical application. Therefore, it is necessary to perform a mismatch detection method on the outputs of DBRN. In this paper, we propose a real-time triple constraint mismatch detection method. The proposed detection method is divided into three modules: the model fitting detection module, the spatial structure detection module, and the distance ratio detection module. In the model fitting detection module, the navigation characteristics of AUVs are used to select the fitting model. In the spatial structure detection module, the proposed method performs the mismatch detection based on the affine transformation relationship between the INS-indicated trajectory and the corresponding matched trajectory. In the distance ratio detection module, we derive the distance ratio constraint between the INS-indicated trajectory and the corresponding matched trajectory. Simulations based on an actual geomagnetic anomaly base map have been performed for the validation of the proposed method.

## 1. Introduction

Autonomous underwater vehicles (AUVs) are widely used in a variety of tasks, including oceanographic surveys, bathymetric data collection in marine and riverine environments, patrol and reconnaissance missions in the military field, and rescue duties [1]. The availability of a precise and robust navigation system is the foundational prerequisite for the vehicle to successfully execute missions [2,3]. The quality of the navigation system not only influences the position errors between the desired path and the executed path, but also affects the outcome of the georeferencing process of the data acquired by the onboard sensors (e.g., the acquisition of terrain and geomagnetic data) [4].

In general, an AUV uses an inertial navigation system (INS) as the primary navigation system [5]. INS is a navigation aid that uses accelerometers and gyroscopes to continuously calculate by dead reckoning the position, the orientation, and the velocity (direction and speed of movement) of a moving object without the need for external references. Nevertheless, INSs have positional errors growth that is unbounded. This effect can be reduced by using accurate acceleration, heading, and velocity sensors, but these sensors cannot be made arbitrarily accurate. During long endurance underwater missions, these inaccuracies become significant [6]. Therefore, other navigation and positioning technologies are adopted by AUVs as auxiliary navigation systems. Above water, the global navigation satellite system (GNSS) is the most commonly used method to compensate for INS-accumulated errors [7]. However, due to the rapid attenuation of higher frequency signals and the unstructured nature of the undersea environment, GNSS signals can only propagate within short distance under water [1]. Database-referenced navigation (DBRN) systems, which use geophysical information such as gravity, magnetic, and terrain data for navigation, hold strong potential as underwater auxiliary navigation systems [8,9]. Take the terrain-referenced navigation (TRN) system (one of the most popular DBRN systems) as an example. TRN obtains a range measurements by using sonar sensors installed on a vehicle. These measurements are matched with a priori digital map of the terrain elevation, to estimate the vehicle position. The principle of gravity-referenced navigation (GRN) and magnetic-referenced navigation (MRN) is the same as TRN, except for the priori digital maps and sensors used. GRN uses gravity anomaly maps and gravimeters as a priori digital maps and sensors. MRN uses geomagnetic anomaly maps and magnetometers as a priori digital maps and sensors. DBRN systems are completely passive and difficult to interfere with; these advantages totally meet the requirements of underwater vehicles [10].

A DBRN system consists of a priori geophysical information database (terrain elevation maps, gravity anomaly maps, etc.), a measurement unit (sonar, gravimeter, etc.), and a navigation algorithm [11,12]. The navigation algorithm is one of the key factors in DBRN [13,14]. There are two types of navigation algorithms in DBRN systems: filtering algorithms and matching algorithms. Filtering algorithms make use of the INS model and the measurement model to construct filters. In DBRN systems, commonly used filtering algorithms are the extended Kalman filter (EKF), the particle filter (PF) and the Rao-Blackwellized particle filter (RBPF) [15,16,17,18]. EKF is more effective for low nonlinear estimation problems. A PF can effectively handle highly nonlinear or non-Gaussian estimation problems. RBPF is a hybrid filter combining EKF and PF. Terrain contour matching (TERCOM) algorithm and iterated closest contour point (ICCP) algorithm are two conventional matching algorithms in DBRN systems [12,19]. TERCOM algorithm, realized via group correlation analysis, possesses better robustness. The ICCP algorithm, which uses rigid transformation to match the multilateral arc, has a high matching accuracy when the initial INS error is small. It should be noted that matching algorithms-based DBRN systems can operate effectively on the premise that the matching area has obvious features. Features can be discrete landmark objects, numerical changes, or texture variations in the digital map. If the features are smooth, the above matching algorithms have a high probability of mismatching [19,20]. Since it is impossible to completely eliminate mismatches, a mismatch detection method after the matching algorithm deserves to be studied.

Mismatch detection methods are widely used in the field of feature points-based image registration. In DBRN systems, the actual trajectory and the DBRN output trajectory are denoted as the original image and the transformed image, respectively, then mismatch detection algorithms in image registration can be used to solve the mismatch detection problem in DBRN systems. Random sample consensus (RANSAC)-based algorithms [21,22,23] and graph transformation matching (GTM)-based algorithms [24,25] are two types of commonly used mismatch detection methods in the field of feature points based image registration. RANSAC is an iterative method to estimate parameters of a mathematical model from a set of observed data that contains outliers, when outliers are to be accorded no influence on the values of the estimates. Therefore, it can be interpreted as an outlier detection method. GTM uses the distribution of matching points and the neighborhood relationship in the local area to detect mismatched points. RANSAC based algorithms and GTM based algorithms can be directly used for detecting mismatched points in DBRN systems. In addition to the above two commonly used types of algorithms, the restricted spatial order constraints (RSOC) algorithm [26] is also a simple and robust outlier detection algorithm. In RSOC, the two-way spatial order constraints are used to determine the candidate outliers, whereas the two decision criteria restrictions are used to confirm which dubious match pairs should be removed. Han et al. [9] proposed an RSOC-based mismatch diagnostic method specifically for an underwater navigation system. The spatial structure constraints and transformation error restrictions are utilized to select outliers in a matched sequence. However, there exists several disadvantages of using these algorithms in DBRN systems. On one hand, these approaches can only detect a certain type of mismatch, RANSAC-based algorithms cannot detect the mismatched point that fits the model, GTM based algorithms and RSOC based algorithms cannot detect the mismatched point with the same structure. On the other hand, applying these approaches requires sampling enough points, which leads to poor real-time performance.

In order to solve the problem of a single detection mode and poor real-time performance, we suggest a real-time triple constraint mismatch detection method. The proposed method, considering the model fitting constraint, spatial structure constraint, and distance ratio constraint, is specifically presented to deal with mismatched point detection problem in matching algorithms-based DBRN systems for AUVs. In model-fitting detection module, the proposed method eliminates points that deviate significantly from the matched trajectory. In the spatial structure detection module, the K-nearest neighbor (KNN) graph of the INS-indicated trajectory and the matched trajectory are established. The affine transformation relationship between the INS-indicated trajectory and the corresponding matched trajectory is used to determine whether the current matched point is an abnormal point. In the distance ratio detection module, the underwater INS error propagation model is established to calculate the distance ratio constraint between the actual trajectory and the INS-indicated trajectory. The matched points beyond the constraint range are regarded as outliers. It should be noticed that the above three detection processes perform mismatch detection point-by-point instead of detecting all mismatched points at one time. These are some important contributions for our work. 

The rest of the paper is organized as follows. The second part briefly describes M-estimator sample consequence (MSAC) and weighted graph transformation matching (WGTM). The INS error propagation model employed in this paper is described in the third part. The fourth part presents the procedure of the proposed method. A comprehensive discussion on the experimental settings and simulation results are provided in the fifth part. Conclusions are given in the last part.

## 2. MSAC and WGTM

### 2.1. RANSAC and MSAC

RANSAC is a robust approach that is used in many applications for extracting shapes and for estimating the model parameters from data that contains outliers. RANSAC classifies data into inliers (data that can be described by the model) and outliers (data that deviate from the normal range of the model or fail to meet the model). It looks for a minimal subset with maximal support (the number of data points that match with the model). RANSAC operates in two steps iteratively to remove outliers: hypothesize and test. First, a minimal subset is randomly selected from the data and the required model parameters are estimated based on the subset. Next, it tests the model against all other data to find inliers. The iteration stops when the probability of obtaining a model with better support than the current best model is below a given threshold. RANSAC can be expressed as calculating the minimum cost function:

(1) $$C=\sum_{i}\rho (e_{i})$$

where $e_{i}$ is the error for the *i*th observation relative to the model, ρ(e) represents the loss function, and:

(2) $$\rho (e)=\{\begin{array}{cc} 0 & \left|e\right|<T\\ \mathrm{constant} & \left|e\right|\geq T \end{array}$$

*T* is the correct error threshold. If *T* is set too high, then the robust estimate can be very poor. 

MSAC [27] extends the boundaries of the loss function in RANSAC; a robust loss function is defined as below:

(3) $$\rho_{2}(e)=\{\begin{array}{ll} e^{2} & e^{2}<T^{2}\\ T^{2} & e^{2}\geq T^{2} \end{array}$$

The advantage of using MSAC in point cloud data analysis has been demonstrated [28]. Choi et al. [29] evaluated the RANSAC family and showed that MSAC is one of the most accurate methods.

### 2.2. GTM and WGTM

GTM assumes two sets of match correspondences between two images: $P=\left\{p_{i}\right\}$ and $Q=\left\{q_{i}\right\}$ of size *N* where $p_{i}$ matches $q_{i}$. The median KNN graph of the two images are $G_{p}=\left\{V_{p},E_{p}\right\}$ and $G_{q}=\left\{V_{q},E_{q}\right\}$, respectively. $G_{p}=\left\{V_{p},E_{p}\right\}$ is created by the following steps: first, define vertex $v_{i}$ for each match correspondence $p_{i}$, such that $V_{p}=\left\{v_{1},v_{2},\ldots ,v_{N}\right\}$; second, a non-directed edge between $v_{i}$ and $v_{j}$ exists when $p_{j}$ is one of the K-nearest neighbors of $p_{i}$, and also, $\left\|p_{i}-p_{j}\right\|\leq \eta$. Here, η is defined by the following expression:

(4) $$\eta =\underset{(l,m)\in V_{P}\times V_{P}}{median}\left\|p_{l}-p_{m}\right\|$$

The adjacency matrix corresponding to $G_{p}$ is denoted with $A_{p}$:

(5) $$A_{p}(i,j)=\{\begin{array}{cc} 1 & (i,j)\in E_{p}\\ 0 & (i,j)\notin E_{p} \end{array}$$

Similarly, the graph $G_{q}$ and the adjacency matrix $A_{q}$ are generated in the same way. If all correspondences were correct, the two graphs would be isomorphic. The residual adjacency matrix $R=\left|A_{p}-A_{q}\right|$ is used to calculate the similarity of two graphs.

GTM completes the detection of mismatched points through iteration. Firstly, an outlier is selected from the matching set by the following expression:

(6) $$j^{out}=\underset{j=1,2,\ldots ,N}{argmax}\sum_{i=1}^{N}R(i,j)$$

Secondly, the outlier and its correspondences are removed, and *N* is decreased by one; thirdly, both KNN graphs are regenerated, and the next iteration is continued. The algorithm stops when $\begin{array}{cc} R(i,j)=0, & \forall i,j \end{array}$.

Instead of relying only on the relationship between adjacent features, WGTM utilizes angular distances between the neighboring features. WGTM can be described by following steps [26].

(1) Like GTM, WGTM also generates two median KNN graphs $G_{p}=\left\{V_{p},E_{p}\right\}$ (with adjacency matrix $A_{p}$) and $G_{q}=\left\{V_{q},E_{q}\right\}$ (with adjacency matrix $A_{q}$) for the sets $P=\left\{p_{i}\right\}$ and $Q=\left\{q_{i}\right\}$. The difference is that the two graphs of $G_{p}$ and $G_{q}$ are directed graphs. A directed edge (i,j) exists when $p_{j}$ is one of the K-nearest neighbors of $p_{i}$, and also, $\left\|p_{i}-p_{j}\right\|\leq \eta$, η is defined by (4). The adjacency matrix is defined by (5).
(2) Find all vertices of $G_{p}$ with at most one edge with other vertices, and remove them and their correspondences from $G_{p}$ and $G_{q}$. Regenerate $G_{p}$ and $G_{q}$. Repeat this process until all vertices of $G_{p}$ have a minimum of two edges.
(3) For the edge that connects $v_{i}$ to $v_{m}$ compute a weight value using the following equation:
  (7) $$W(i,m)=\left|\arccos\left(\frac{(p_{m}-p_{i})\cdot \left(Rot(\theta (k_{\min},i))(q_{m}-q_{i})\right)}{\left\|p_{m}-p_{i}\right\|\left\|q_{m}-q_{i}\right\|}\right)\right|$$
  where:
  (8) $$Rot(\theta (k_{\min},i))=\left[\begin{array}{cc} \cos(\theta (k_{\min},i)) & -\sin(\theta (k_{\min},i))\\ \sin(\theta (k_{\min},i)) & \cos(\theta (k_{\min},i)) \end{array}\right]$$
  Here, $k_{\min}$ represents the optimal rotation angle between each pair of matches. $p_{i}$ and $p_{m}$ are the 2D vectors of the image coordinates for vertices $v_{i}$ and $v_{m}$.
(4) For each vertex $v_{i}$ in $V_{p}$, find the percentage of edges that are connected to $v_{i}$ with their correspondences connected to $v_{i}^{\prime }$ in $V_{q}$. The weight value is set to be π if the percentage is smaller than 50%.
(5) For each vertex $v_{i}$ in $V_{p}$, compute the mean of all weights by (9). Remove the vertex corresponding to the maximum value of w and all of its corresponding vertices from P and Q:
  (9) $$w(i)=\underset{\forall j,(i,j)\in E_{p}}{\mathrm{mean}}(W(i,j))$$
(6) If the maximum value of w is less than π, and the change in the mean value of w(i) is less than the threshold, the iteration is terminated; otherwise, it goes to the next iteration.

## 3. The INS Error Propagation Model

An INS is available in this paper; hence, an INS error propagation model [30] is employed in this paper. The INS error propagation model in [30] is modified in this paper, since the n-reference frame adopted in underwater vehicles (north, east and down reference frames) is different from the n-reference frame adopted in [30] (east, north, and up reference frames).

Let $\alpha_{z}$, $\alpha_{y}$, $\alpha_{x}$ be the three rotational angles from n-reference frame (ideal mathematics platform) to the n′-reference frame (actual mathematics platform), $\alpha =\left[\begin{array}{ccc} \alpha_{x} & \alpha_{y} & \alpha_{z} \end{array}\right]$; the rotation matrixes can be expressed as:

(10) $$\begin{array}{l} C_{\alpha_{z}}=\left[\begin{array}{ccc} \cos\alpha_{z} & -\sin\alpha_{z} & 0\\ \sin\alpha_{z} & \cos\alpha_{z} & 0\\ 0 & 0 & 1 \end{array}\right]\\ C_{\alpha_{y}}=\left[\begin{array}{ccc} \cos\alpha_{y} & 0 & -\sin\alpha_{y}\\ 0 & 1 & 0\\ \sin\alpha_{y} & 0 & \cos\alpha_{y} \end{array}\right]\\ C_{\alpha_{x}}=\left[\begin{array}{ccc} 1 & 0 & 0\\ 0 & \cos\alpha_{x} & \sin\alpha_{x}\\ 0 & -\sin\alpha_{x} & \cos\alpha_{x} \end{array}\right] \end{array}$$

Then, the coordinate transformation matrix from the n-reference frame to the n′-reference frame is derived:

(11) $$1C_{n}^{n\prime }=C_{\alpha_{x}}C_{\alpha_{y}}C_{\alpha_{z}}$$

Denoting with $\omega_{\mathrm{nn}\prime }^{n\prime }$ the relative angular velocity of the n′-reference frame in the n-reference frame:

(12) $$\omega_{\mathrm{nn}\prime }^{n\prime }=C_{\alpha_{x}}C_{\alpha_{y}}\left[\begin{array}{c} 0\\ 0\\ {\dot{\alpha }}_{z} \end{array}\right]+C_{\alpha_{x}}\left[\begin{array}{c} 0\\ {\dot{\alpha }}_{y}\\ 0 \end{array}\right]+\left[\begin{array}{c} {\dot{\alpha }}_{x}\\ 0\\ 0 \end{array}\right]=C_{\omega }\left[\begin{array}{c} {\dot{\alpha }}_{x}\\ {\dot{\alpha }}_{y}\\ {\dot{\alpha }}_{z} \end{array}\right]$$

Hence, the differential equation of Euler platform error angles can be expressed as follows:

(13) $$\dot{\alpha }=C_{\omega }^{-1}\omega_{\mathrm{nn}\prime }^{n\prime }$$

(14) $$\begin{array}{c} C_{\omega }=\left[\begin{array}{ccc} 1 & 0 & -\sin\alpha_{y}\\ 0 & \cos\alpha_{x} & \sin\alpha_{x}\cos\alpha_{y}\\ 0 & -\sin\alpha_{x} & \cos\alpha_{x}\cos\alpha_{y} \end{array}\right]\\ C_{\omega }^{-1}=\frac{1}{\cos\alpha_{y}}\left[\begin{array}{ccc} \cos\alpha_{y} & \sin\alpha_{x}\sin\alpha_{y} & \cos\alpha_{x}\sin\alpha_{y}\\ 0 & \cos\alpha_{x}\cos\alpha_{y} & -\sin\alpha_{x}\cos\alpha_{y}\\ 0 & \sin\alpha_{x} & \cos\alpha_{x} \end{array}\right] \end{array}$$

Denoting with $\omega_{\mathrm{ie}}^{n}$ the rotational angular velocity of the earth, *L* and *h* are the latitude and depth, respectively. *R*_M_ is the local radius of the curvature in the meridian, and *R*_N_ is the local radius of the curvature in the prime vertical. $\hat{L}=L+\delta L$, $\hat{h}=h+\delta h$. δL and δh are both slight errors. The following equations have been derived:

(15) $$\omega_{\mathrm{ie}}^{n}={\left[\begin{array}{ccc} \omega_{\mathrm{ie}}\cos L & 0 & -\omega_{\mathrm{ie}}\sin L \end{array}\right]}^{T}$$

(16) $$\omega_{\mathrm{en}}^{n}={\left[\begin{array}{ccc} \frac{v_{E}^{n}}{R_{N}-h} & -\frac{v_{N}^{n}}{R_{M}-h} & -\frac{v_{E}^{n}}{R_{N}-h}\tan L \end{array}\right]}^{T}$$

(17) $$\delta \omega_{\mathrm{ie}}^{n}=\left[\begin{array}{c} -\omega_{\mathrm{ie}}\sin\hat{L}\delta L\\ 0\\ -\omega_{\mathrm{ie}}\cos\hat{L}\delta L \end{array}\right]$$

(18) $$\delta \omega_{\mathrm{en}}^{n}=\left[\begin{array}{c} \delta v_{E}^{n}/\left({\hat{R}}_{N}-\hat{h}\right)\\ -\delta v_{N}^{n}/\left({\hat{R}}_{M}-\hat{h}\right)\\ -\left(\tan\hat{L}\delta v_{E}^{n}+{\hat{v}}_{E}^{n}\sec^{2}\hat{L}\delta L\right)/\left({\hat{R}}_{N}-\hat{h}\right) \end{array}\right]$$

On the basis of formulas above, the INS error propagation model that is used in the underwater environment have been derived [30]:

(19) $$\{\begin{array}{l} \delta \dot{L}=\frac{\delta v_{N}^{n}}{R_{M}-h}+\delta h\frac{v_{N}^{n}}{{\left(R_{M}-h\right)}^{2}}\\ \delta \dot{\lambda }=\frac{\delta v_{E}^{n}}{R_{N}-h}\sec L+\delta L\frac{v_{E}^{n}}{R_{N}-h}\tan L\sec L+\delta h\frac{v_{E}^{n}\sec L}{{\left(R_{N}-h\right)}^{2}}\\ \delta \dot{h}=\delta V_{D}\\ \dot{\alpha }=C_{\omega }^{-1}\left[\left(I-C_{n}^{n\prime }\right){\hat{\omega }}_{\mathrm{in}}^{n}+C_{n}^{n\prime }\delta \omega_{\mathrm{in}}^{n}-C_{b}^{n\prime }\varepsilon^{b}\right]-C_{\omega }^{-1}C_{b}^{n\prime }w_{g}^{b}\\ \begin{array}{ll} \delta {\dot{v}}^{n} & =\left[I-{\left(C_{n}^{n\prime }\right)}^{T}\right]C_{b}^{n\prime }{\hat{f}}_{\mathrm{sf}}^{b}+{\left(C_{n}^{n\prime }\right)}^{T}C_{b}^{n\prime }\nabla^{b}-\left(2\delta \omega_{\mathrm{ie}}^{n}+\delta \omega_{\mathrm{en}}^{n}\right)\times \left({\hat{v}}^{n}-\delta v^{n}\right)\\ & -\left(2{\hat{\omega }}_{\mathrm{ie}}^{n}+{\hat{\omega }}_{\mathrm{en}}^{n}\right)\times \delta v^{n}+{\left(C_{n}^{n\prime }\right)}^{T}C_{b}^{n\prime }w_{a}^{b} \end{array}\\ {\dot{\varepsilon }}^{b}=0\\ {\dot{\nabla }}^{b}=0 \end{array}$$

where $\varepsilon^{b}$ and $w_{g}^{b}$ are the constant bias and zero mean Gaussian white noise of the gyroscope, respectively, and $\nabla^{b}$ and $w_{a}^{b}$ are the constant bias and zero mean Gaussian white noise of the accelerometer, respectively. 

## 4. The Real-Time Triple Constraint Mismatch Detection Method

In DBRN, the matching process involves a digital base map. The resolution of the base map and the matching features greatly affect the matching accuracy. In the case of a base map with a large resolution, or in the case of the smooth matching features, mismatch has a high probability of occurrence. Therefore, it is necessary to develop a mismatch detection method for matching algorithms-based DBRN systems. 

As the primary navigation system of AUV, INS has the characteristics of short-term high precision. INS can give continuous accurate positions to begin with, but the accumulated position errors are unacceptable after a long period underway. The position error of INS increases slowly with time, but it does not increase suddenly and rapidly in a short period, which means that in a period of time, the relative positions between the INS-indicated positions and the corresponding actual positions can be regarded as being accurate and reliable. The shape of the INS-indicated trajectory is supposed to be similar to the corresponding actual vehicle trajectory [31,32]. Therefore, an INS-indicated trajectory and the corresponding matched trajectory can be used as inputs for WGTM to perform mismatch detection. In addition, considering that AUVs are able to maintain a direct (linear) trajectory during navigation underwater [5], the matched points can be fitted by using a linear model. It is thus possible to detect mismatched points using MSAC. However, the direct use of WGTM and MSAC on AUV for mismatch detection has the following drawbacks:

(1) MSAC and WGTM have poor real-time performance. These two algorithms eliminate all of the outliers by iteration. Conversely, in a navigation system, the most important thing is to judge whether the current position is mismatched.
(2) MSAC cannot detect the mismatched point that fits the model, and WGTM cannot detect the mismatched point with the same structure.
(3) Both MSAC and WGTM do not take into account the distance constraint between the sampling points.

**Figure 1:**
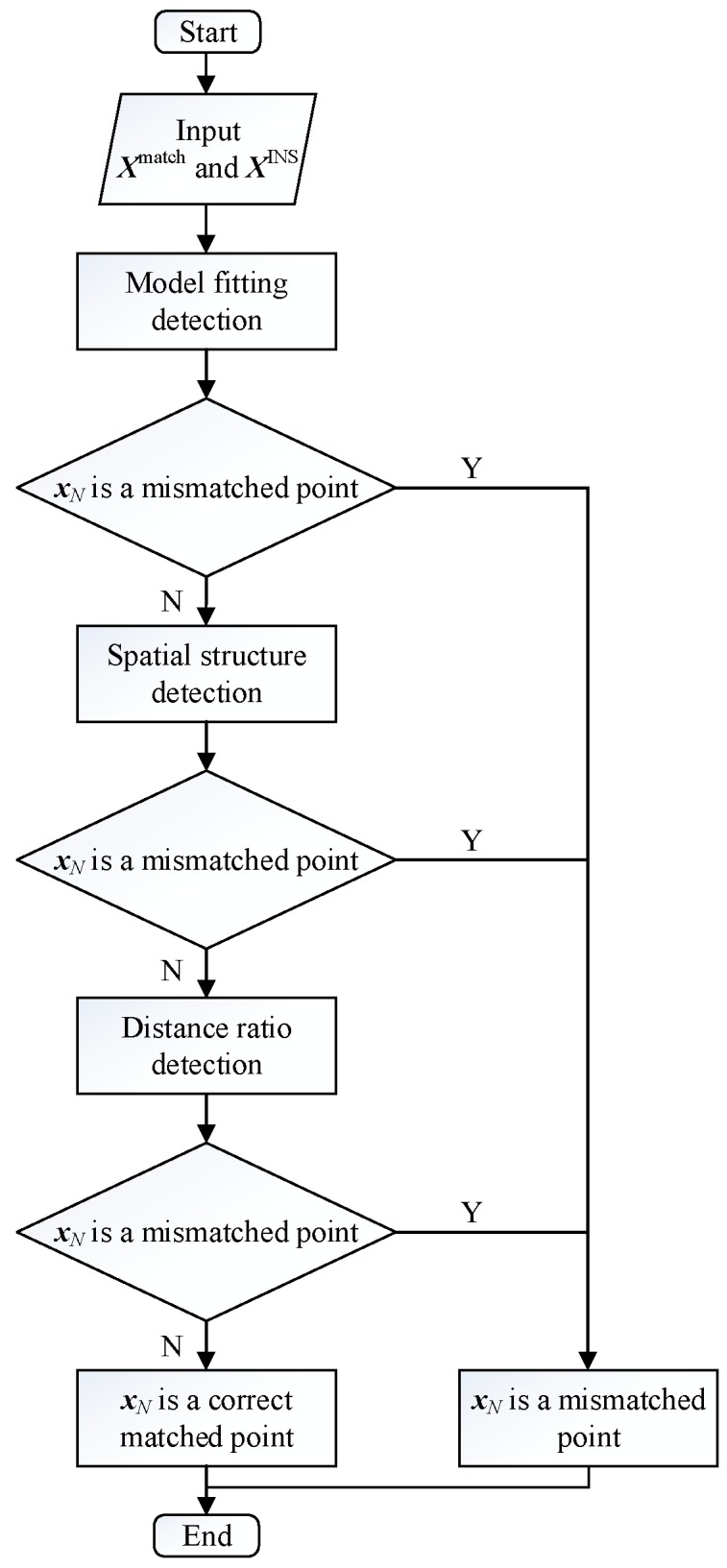
The flow of the proposed mismatch detection method.

In view of the limitations of MSAC and WGTM applied on AUV, this paper proposes a real-time triple constraint mismatch detection method. The real-time triple constraint mismatch detection method is divided into three modules: the model fitting detection module, the spatial structure detection module, and the distance ratio detection module. The proposed method changes multi-point simultaneous detection into real-time detection of the current point. Due to the short-term high-precision of INS, the INS-indicated trajectory can be approximated as the actual trajectory during the initial period of time (assuming that the constant drift and random drift of gyro are both 0.02 °/hr, the accelerometer’s constant bias and random bias are both 100 µg, and then the position error of INS after 10 min of navigation is about 0.2′, which is a resolution that most of the digital base map cannot reach). This feature will be used to select the input trajectory. In this paper, MSAC and WGTM are simplified to make it suitable for AUV real-time mismatch detection. In addition, the distance ratio constraint between the INS-indicated trajectory and the corresponding matched trajectory is established according to the INS error propagation model. The flow of the proposed method is shown in Figure 1. 

### 4.1. Model Fitting Detection

Based on MSAC, the proposed detection method detects the fitting degree of the current matching result with the optimal linear model. In the model fitting detection module, $X^{\mathrm{match}}=\left\{x_{1}^{\mathrm{match}},x_{2}^{\mathrm{match}},\ldots ,x_{N}^{\mathrm{match}}\right\}$ are the input point sets. Here $x_{N}^{\mathrm{match}}$ is the current point (the output of matching algorithm at the current moment), and $x_{i}^{\mathrm{match}}$ (i={1,2,…,N−1}) are N−1 correct matched points closest to $x_{N}^{\mathrm{match}}$. If there are fewer than N−1 points successfully matched before $x_{N}^{\mathrm{match}}$, the remaining points are supplemented by the INS-indicated position that can be considered the actual position. As mentioned before, considering the short-term high-precision characteristics of INS, as long as the accumulated position error calculated by (19) is smaller than one grid, the INS-indicated position can be regarded as the actual position. Define $e_{x_{i}^{\mathrm{match}}}$ as the Euclidean distance between $x_{i}^{\mathrm{match}}$ and the current linear model ***M***. Functions (1) and (3) are used to generate the value of cost function *C**_M_***. ***M*** with the largest number of inliers, and the smallest value of *C**_M_*** is the best linear model ***M***_best_. In practical experiments, the correct error threshold *T* is set to three grids (found empirically). Finally, the error $e_{x_{i}^{\mathrm{match}}}$ is calculated for each point in $X^{\mathrm{match}}$ relative to ***M***_best_. If $x_{N}^{\mathrm{match}}$ is an outlier and $e_{x_{N}^{\mathrm{match}}}$ is the largest error among all points in $X^{\mathrm{match}}$, then $x_{N}^{\mathrm{match}}$ is a mismatched point. The pseudo-code of model fitting detection is shown in Algorithm 1.

**Algorithm 1.** Model fitting detection**Begin** 1. select input point set $X^{\mathrm{match}}$ 2. initialize $N_{M_{\mathrm{best}}}^{\mathrm{inliers}}=0$ 3. for *I* = 1 to N−1 4.  for *j* = 2 to *N*    calculate the parameters of the linear model ***M*** according to $x_{i}^{\mathrm{match}}$ and $x_{j}^{\mathrm{match}}$    compute $N_{M}^{\mathrm{inliers}}$ (the number of inliers) in $X^{\mathrm{match}}$ based on ***M*** and $\rho_{2}$    calculate the cost function *C**_M_***    if $N_{M}^{\mathrm{inliers}}>N_{M_{\mathrm{best}}}^{\mathrm{inliers}}$ or ($N_{M}^{\mathrm{inliers}}=N_{M_{\mathrm{best}}}^{\mathrm{inliers}}$ and $C_{M}<C_{M_{\mathrm{best}}}$)     $M_{\mathrm{best}}=M$    end if   end for *j*  end for *i* 5. if $x_{N}^{\mathrm{match}}$ is an outlier and $e_{x_{i}^{\mathrm{match}}}^{\max}=e_{x_{N}^{\mathrm{match}}}$, i={1,2,…,N}, $x_{N}^{\mathrm{match}}$ is a mismatched point
**End**


### 4.2. Spatial Structure Detection

The shape of the INS-indicated trajectory is supposed to be similar to the actual vehicle trajectory. In other words, the INS-indicated trajectory has a similar spatial structure to the corresponding actual trajectory. In the spatial structure detection module, the WGTM algorithm is simplified to make it more suitable for handling mismatch detection problems in matching algorithms-based DBRN systems. 

In image processing, some of the points go through dramatic changes, and they therefore do not retain the invariance property with respect to rotation or scale under large perspective transformation. This problem does not exist in the mismatch detection problem in navigation systems, because the rotation angle between the INS-indicated trajectory and the corresponding actual trajectory is small. Therefore, the second step in WGTM is removed. In addition, the proposed method simplifies the computation by using the rotation angle between the INS-indicated trajectory and the corresponding matched trajectory as the optimal rotation angle. Let $X^{\mathrm{INS}}=\left\{x_{1}^{\mathrm{INS}},x_{2}^{\mathrm{INS}},\ldots ,x_{N}^{\mathrm{INS}}\right\}$ be the INS-indicated trajectory, and let $X^{\mathrm{match}}=\left\{x_{1}^{\mathrm{match}},x_{2}^{\mathrm{match}},\ldots ,x_{N}^{\mathrm{match}}\right\}$ be the corresponding matched trajectory. Denoting with ***Q*** the covariance matrix:

(20) $$Q=\left[\begin{array}{cc} Q_{11} & Q_{12}\\ Q_{21} & Q_{22} \end{array}\right]=\sum_{k=1}^{N}\left(x_{k}^{\mathrm{INS}}-{\tilde{x}}^{\mathrm{INS}}\right){\left(x_{k}^{\mathrm{match}}-{\tilde{x}}^{\mathrm{match}}\right)}^{T}$$

where ${\tilde{x}}^{\mathrm{INS}}$ and ${\tilde{x}}^{\mathrm{match}}$ are, respectively, the average values of all position vectors in $X^{\mathrm{INS}}$ and $X^{\mathrm{match}}$. The eigenvalues of ***Q*** and the rotation angle from $X^{\mathrm{INS}}$ to $X^{\mathrm{match}}$ are calculated through a quaternion algorithm [33]:

(21) $$\begin{array}{l} \lambda_{1,2}=\pm {\left[{\left(Q_{11}+Q_{22}\right)}^{2}+{\left(Q_{21}-Q_{12}\right)}^{2}\right]}^{1/2}\\ \lambda_{3,4}=\pm {\left[{\left(Q_{11}-Q_{22}\right)}^{2}+{\left(Q_{21}+Q_{12}\right)}^{2}\right]}^{1/2} \end{array}$$

(22) $$tg(\frac{\tau }{2})=(S_{11}+S_{22}-\lambda_{m})/(S_{12}-S_{21})$$

where $\lambda_{\mu }$(μ={1,2,3,4}) are four eigenvalues of ***Q***, and $\lambda_{m}$ is the maximum eigenvalue. τ is the rotation angle from $X^{\mathrm{INS}}$ to $X^{\mathrm{match}}$. The spatial structure detection module can be described by the following steps.

(1) Two median KNN graphs $G^{\mathrm{INS}}=\left\{V^{\mathrm{INS}},E^{\mathrm{INS}}\right\}$ (with the adjacency matrix $A^{\mathrm{INS}}$) and $G^{\mathrm{match}}=\left\{V^{\mathrm{match}},E^{\mathrm{match}}\right\}$ (with adjacency matrix $A^{\mathrm{match}}$) are generated for the trajectories $X^{\mathrm{INS}}=\left\{x_{1}^{\mathrm{INS}},x_{2}^{\mathrm{INS}},\ldots ,x_{N}^{\mathrm{INS}}\right\}$ and $X^{\mathrm{match}}=\left\{x_{1}^{\mathrm{match}},x_{2}^{\mathrm{match}},\ldots ,x_{N}^{\mathrm{match}}\right\}$. A directed edge (i,j) exists when $x_{j}$ is one of the K-nearest neighbors of $x_{i}$ and also $\left\|x_{i}-x_{j}\right\|\leq \eta$, η is defined by (4). The adjacency matrix is defined by (5).
(2) For the edge that connects $v_{i}^{\mathrm{match}}$ to $v_{m}^{\mathrm{match}}$ compute a weight value using the following equation:
  (23) $$W(i,m)=\left|\arccos\left(\frac{(x_{m}^{\mathrm{INS}}-x_{i}^{\mathrm{INS}})\cdot \left(R_{\tau }(x_{m}^{\mathrm{match}}-x_{i}^{\mathrm{match}})\right)}{\left\|x_{m}^{\mathrm{INS}}-x_{i}^{\mathrm{INS}}\right\|\left\|x_{m}^{\mathrm{match}}-x_{i}^{\mathrm{match}}\right\|}\right)\right|$$
  where:
  (24) $$R_{\tau }=\left[\begin{array}{cc} \cos\tau & -\sin\tau \\ \sin\tau & \cos\tau \end{array}\right]$$
  Here, τ represents the optimal rotation angle calculated by (22). $R_{\tau }$ is the corresponding rotation matrix.
(3) For vertex $v_{N}^{\mathrm{match}}$, find the percentage of edges that are connected to $v_{N}^{\mathrm{match}}$, with their correspondences connected to $v_{N}^{\mathrm{INS}}$. If the percentage is smaller than 50%, the weight value of all different edges should be replaced by π.
(4) For each vertex $v_{i}^{\mathrm{match}}$, compute the mean of all weights by the following expression:
  (25) $$w(i)=\underset{\forall j,(i,j)\in E^{\mathrm{match}}}{\mathrm{mean}}(W(i,j))$$
(5) If the maximum value of w is not w(N), $x_{N}$ pass the spatial structure detection; otherwise, set:
  (26) $$\mu_{\mathrm{old}}=\underset{\forall i}{\mathrm{mean}}(w(i))$$
  and let $X^{\mathrm{INS}}=\left\{x_{1}^{\mathrm{INS}},x_{2}^{\mathrm{INS}},\ldots ,x_{N-1}^{\mathrm{INS}}\right\}$ and $X^{\mathrm{match}}=\left\{x_{1}^{\mathrm{match}},x_{2}^{\mathrm{match}},\ldots ,x_{N-1}^{\mathrm{match}}\right\}$. Repeat step (1) to step (4), and $\mu_{\mathrm{new}}$ is calculated by (26). If $\left|\mu_{\mathrm{new}}-\mu_{\mathrm{old}}\right|<\varepsilon$, $x_{N}$ passes the spatial structure detection; otherwise, $x_{N}$ is a mismatched point. The value of ε is set to 0.01 (found empirically) for all of the results presented in this work. The pseudo-code of the spatial structure detection is shown in Algorithm 2.

**Algorithm 2.** Spatial Structure Detection**Begin:** 1. select input point sets $X^{\mathrm{INS}}=\left\{x_{i}^{\mathrm{INS}}\right\}$ and $X^{\mathrm{match}}=\left\{x_{i}^{\mathrm{match}}\right\}$, i={1,2,…,N} 2. create $G^{\mathrm{INS}}$, $G^{\mathrm{match}}$, $A^{\mathrm{INS}}$, $A^{\mathrm{match}}$ 3. compute *W* 4. $\forall (i,j)\in E^{\mathrm{match}}$, compute $w(i)=\underset{}{\mathrm{mean}}(W(i,j))$ 5. if max(w(i))≠w(N)  $x_{N}$ pass the spatial structure detection else  $\mu_{\mathrm{old}}=\underset{\forall i}{\mathrm{mean}}(w(i))$  delete $x_{N}$ and regenerate $X^{\mathrm{INS}}=\left\{x_{i}^{\mathrm{INS}}\right\}$, $X^{\mathrm{match}}=\left\{x_{i}^{\mathrm{match}}\right\}$, i={1,2,…,N−1}  repeat steps 1 to 4, calculate $\mu_{\mathrm{new}}$  if $\left|\mu_{\mathrm{new}}-\mu_{\mathrm{old}}\right|<\varepsilon$   $x_{N}$ pass the spatial structure detection  else   $x_{N}$ is a mismatched point  end if end if
**End**


### 4.3. Distance Ratio Detection

There is a scaling error between the INS-indicated trajectory and the corresponding actual trajectory [34]. To better describe the scaling error, let $X^{\mathrm{INS}}=\left\{x_{1}^{\mathrm{INS}},x_{2}^{\mathrm{INS}},\ldots ,x_{N}^{\mathrm{INS}}\right\}$ be the INS-indicated trajectory and let $X^{\mathrm{actual}}=\left\{x_{1}^{\mathrm{actual}},x_{2}^{\mathrm{actual}},\ldots ,x_{N}^{\mathrm{actual}}\right\}$ be the corresponding actual trajectory. The INS-indicated distance and the actual distance in a short period are expressed by the following equations:

(27) $$d_{k-1,k}^{\mathrm{INS}}=\frac{\left\|v_{k-1}^{\mathrm{INS}}+v_{k}^{\mathrm{INS}}\right\|}{2}\cdot \Delta t_{k-1,k}$$

(28) $$d_{k-1,k}^{\mathrm{actual}}\approx \frac{\left\|v_{k-1}^{\mathrm{actual}}+v_{k}^{\mathrm{actual}}\right\|}{2}\cdot \Delta t_{k-1,k}$$

$d_{k-1,k}^{\mathrm{INS}}$ and $d_{k-1,k}^{\mathrm{actual}}$ are, respectively, the INS-indicated distance and the actual distance between $x_{k-1}$ and $x_{k}$, respectively; k∈{2,3,…,N}. $v_{k}^{\mathrm{INS}}$ and $v_{k}^{\mathrm{actual}}$ are the INS-indicated linear velocity and the actual linear velocity, respectively, of $x_{k}$. $\Delta t_{k-1,k}$ is the time interval between $x_{k-1}$ and $x_{k}$. Then, the relationship between $d_{k-1,k}^{\mathrm{INS}}$ and $d_{k-1,k}^{\mathrm{actual}}$ is derived:

(29) $$\frac{d_{k-1,k}^{\mathrm{actual}}}{d_{k-1,k}^{\mathrm{INS}}}=\frac{\left\|v_{k-1}^{\mathrm{actual}}+v_{k}^{\mathrm{actual}}\right\|}{\left\|v_{k-1}^{\mathrm{INS}}+v_{k}^{\mathrm{INS}}\right\|}$$

where $v_{k}^{\mathrm{INS}}$ is described by an equation in the form:

(30) $$v_{k}^{\mathrm{INS}}=v_{k}^{\mathrm{actual}}+\delta v_{k}$$

Here $\delta v_{k}$ is the INS-accumulated velocity error of $x_{k}$. Accordingly, (29) is developed into the following form:

(31) $$\frac{d_{k-1,k}^{\mathrm{actual}}}{d_{k-1,k}^{\mathrm{INS}}}=\frac{\left\|v_{k-1}^{\mathrm{INS}}+v_{k}^{\mathrm{INS}}-\delta v_{k-1}-\delta v_{k}\right\|}{\left\|v_{k-1}^{\mathrm{INS}}+v_{k}^{\mathrm{INS}}\right\|}$$

Through (19) and (31), the range of $\frac{d_{k-1,k}^{\mathrm{actual}}}{d_{k-1,k}^{\mathrm{INS}}}$ during the entire sailing period can be estimated. Define $S_{\min}$ as the lower bound of the range and $S_{\max}$ as the upper bound of the range. The range can be expressed by the following expression:

(32) $$\frac{d_{k-1,k}^{\mathrm{actual}}}{d_{k-1,k}^{\mathrm{INS}}}\in \left(S_{\min},S_{\max}\right)$$

Based on the premise described above, for a matched trajectory $X^{\mathrm{match}}$, if $X^{\mathrm{match}}$ is a correctly matched trajectory, $\frac{d_{k-1,k}^{\mathrm{match}}}{d_{k-1,k}^{\mathrm{INS}}}$ should meet the range described in (32), k={1,2,…,N}. Here $d_{k-1,k}^{\mathrm{match}}$ is the distance between $x_{k-1}^{\mathrm{match}}$ and $x_{k}^{\mathrm{match}}$. The pseudo-code of the distance ratio detection is shown in Algorithm 3. 

**Algorithm 3.** Distance ratio detection**Begin:** 1. select input point sets $X^{\mathrm{INS}}=\left\{x_{i}^{\mathrm{INS}}\right\}$ and $X^{\mathrm{match}}=\left\{x_{i}^{\mathrm{match}}\right\}$, i={1,2,…,N} 2. compute $\frac{d_{N-1,N}^{\mathrm{match}}}{d_{N-1,N}^{\mathrm{INS}}}$ 3. if $S_{\min}\leq \frac{d_{N-1,N}^{\mathrm{match}}}{d_{N-1,N}^{\mathrm{INS}}}\leq S_{\max}$  $x_{N}$ passes the distance ratio detection else  $x_{N}$ is a mismatched point end if
**End**


## 5. Simulation and Analysis

In order to test the feasibility of our approach, numerous simulation experiments have been performed. Firstly, an INS-indicated trajectory was generated. Secondly, the vector iterated closest contour point (VICCP) algorithm [34] was conducted to obtain a matched trajectory. Thirdly, the proposed mismatch detection method was performed. Fourthly, we used the RSOC-based mismatch diagnostic [9] algorithm as a reference, comparing the results of the RSOC-based mismatch diagnostic algorithm with the proposed algorithm. Finally, we analyzed the impact of thresholds on the proposed method.

### 5.1. Simulation of INS

**Figure 2:**
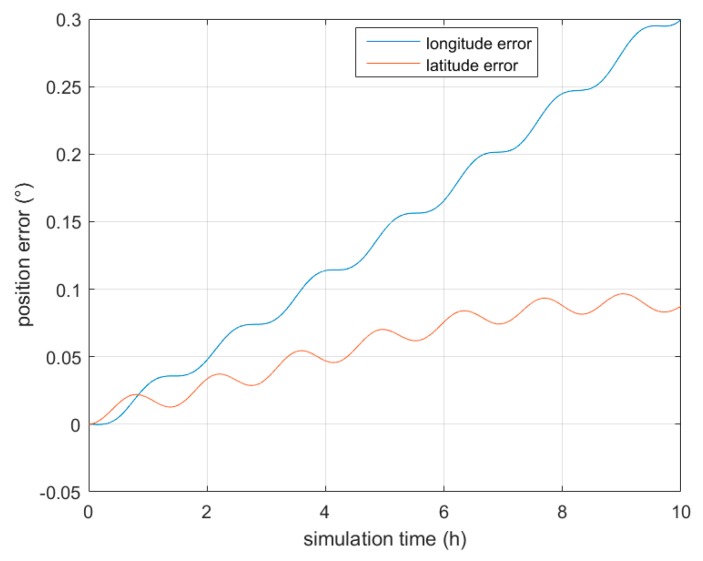
The INS position error.

An INS-indicated trajectory starting from (156.4°E, 19.7°N) was generated, and the INS-relevant parameters are listed in Table 1. The INS position error is shown in Figure 2. As seen from Figure 2, the position error of the generated trajectory exhibited a Schuler oscillation of 84.4 min.

### 5.2. Simulation of the Proposed Algorithm

**Figure 3:**
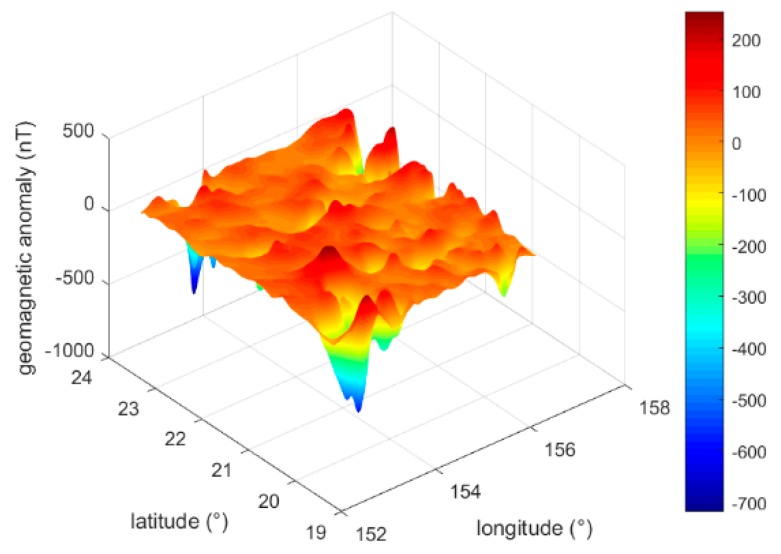
3D map of the geomagnetic anomaly data.

To perform the mismatch detection method presented in this work, in addition to the INS-indicated trajectory, a matched trajectory is required. In the simulation experiment, we used the VICCP algorithm as the matching algorithm. The EMAG2 model [35] was used to calculate the geomagnetic anomaly in the area from (152.4°E, 19.5°N) to (156.6°E, 23.7°N). After interpolation, the grid step was converted to 0.3′. The 3D maps of the geomagnetic anomaly data are shown in Figure 3. The geomagnetic anomaly-relevant parameters are shown in Table 2.

**Figure 4:**
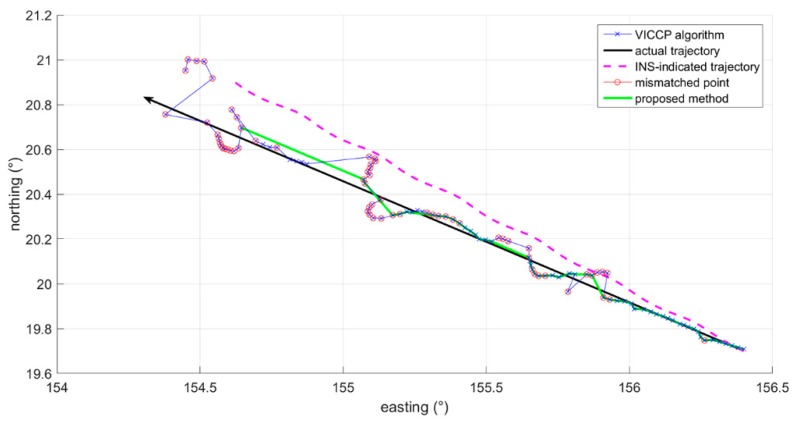
Matching results within nine hours.

**Figure 5:**
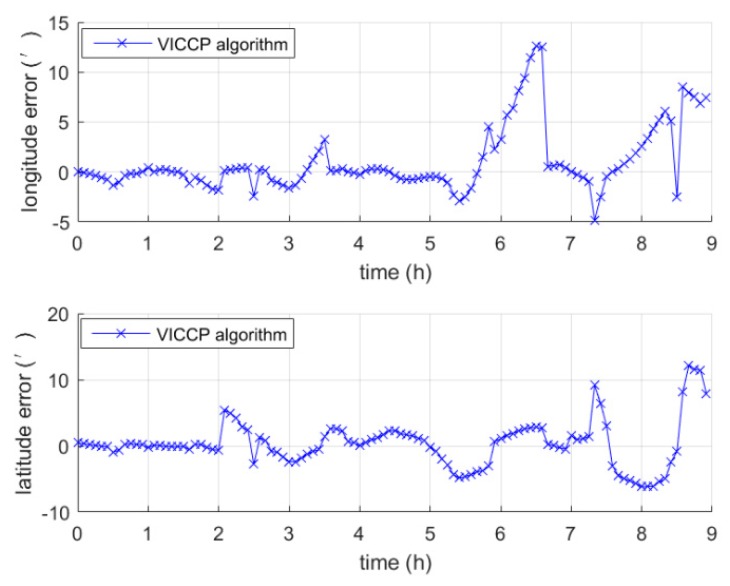
Longitude error and latitude error within nine hours.

Table 3 shows the configuration of the VICCP algorithm. After the INS-indicated trajectory and the corresponding matched trajectory calculated by VICCP are gained, their position information is delivered to the proposed method. *T* and ε in the proposed method are set to 3 grid and 0.01, respectively. Figure 4 shows the INS-indicated trajectory, the matched trajectory calculated by VICCP, and the trajectory after performing the proposed mismatch detection method within nine hours. In Figure 4, the red cycle in VICCP trajectory represents the mismatched point, which is defined as the point where the position error is greater than five grids. The black arrow in Figure 4 indicates the direction of navigation. Obviously, compared to the VICCP trajectory in Figure 4, the matched trajectory obtained by the proposed method was significantly closer to the actual trajectory. Figure 5 indicates the longitude error and latitude error of each matching point. At the beginning, the VICCP algorithm could hold a highly successful matching rate. However, with the initial position error increase, the successful matching rate decreased significantly. 

### 5.3. Comparison between the RSOC-Based Mismatch Diagnostic Algorithm and the Proposed Method

**Figure 6:**
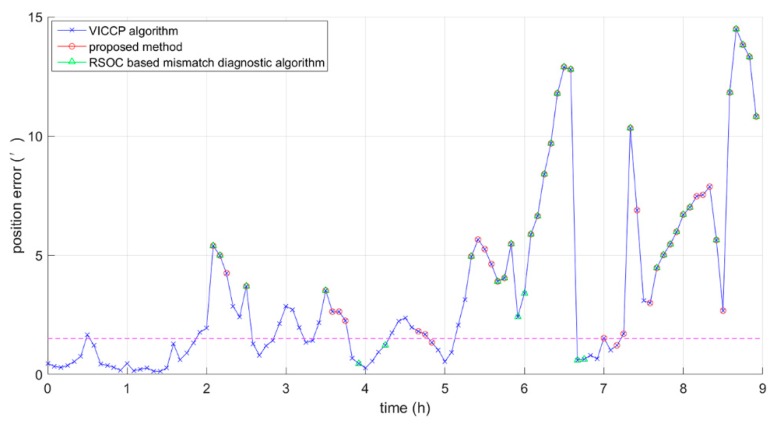
Mismatch detection results of the restricted spatial order constraints (RSOC)-based mismatch diagnostic algorithm and the proposed method.

In the comparative experiment of the RSOC-based mismatch diagnostic algorithm and the proposed method, we used the RSOC-based mismatch diagnostic algorithm and the proposed method to detect the same trajectory. *a* and *b* in the RSOC based mismatch diagnostic algorithm were set to 1 grid and 0.5 grid, respectively. Figure 6 shows the results of the two detection methods, the green circles represent the detected results obtained by the RSOC-based mismatch diagnostic algorithm, and the red circles represent the detected results obtained by the proposed method. In Figure 6, the pink dotted line is the boundary between the correct match and the mismatch, the mismatched points are above the dotted line, and correct matched points are below the dotted line. In order to better evaluate the proposed method, the statistical results of the two methods are given in Table 4. In Table 4, a matching result is considered to be a successful match if the position error is within 5 grids (1.5′). The results of the two mismatch detection methods include correct detection (points with a position error of greater than 5 grids) and error detection (points with a position error of less than 5 grids). The proportion of the correct detection in the detection results can measure the performance of a mismatch detection algorithm. In addition, the detection results do not necessarily include all of the mismatched points on the trajectory. The proportion of the correct detection results in all mismatched points was also used to measure the performance of the algorithm. Column *CD* (Correct Detection) represents the number of points in the detection results where the position error is greater than five grids. The column *TN* (total number) stands for the total number of points obtained by the corresponding detection algorithm. *TN*’ is defined as the total number of the points with position errors of greater than five grids on the trajectory. *CR* (correctness rate) and *DR* (detection rate) are defined by the following expressions:

(33) $$CR=\frac{CD}{TN}$$

(34) $$DR=\frac{CD}{TN^{\prime }}$$

It can be concluded from Figure 6 and Table 4 that the proposed method can detect more mismatched points than the RSOC-based mismatch diagnostic algorithm. Moreover, since the proposed method can perform mismatch detection point-by-point, the proposed method can perform a mismatch detection every time the current location is updated. However, the RSOC-based mismatch diagnostic algorithm performs a mismatch detection sequence by sequence, and it cannot perform mismatch detection immediately when the location is updated. Therefore, the proposed method can detect the matching result in real time. 

### 5.4. Influence of the Threshold Value

In the proposed method, *T* and ε are two threshold values that need to be set. The impact on the detection results of the threshold value is discussed in this part. The statistic results obtained by the different thresholds are shown in Table 5. 

It was determined that the smaller the values of *T* and ε, the more mismatched points are detected, and the greater the probability of false detection. The larger the values of *T* and ε, the smaller the role of the model-fitting detection module and the spatial structure detection module in the proposed mismatch detection method. 

The settings of the threshold values depended on the resolution of the base map and the accuracy of the INS. Under the simulation conditions of this paper, when *T* was set to 3 grid~5 grid and ε was set to 0.01~0.05, the detection results varied slightly.

## 6. Conclusions

In this paper, a real-time mismatch detection method for DBRN is proposed. The proposed method consists of model-fitting detection module, spatial structure detection module, and a distance ratio detection module. In the model-fitting detection module, we used the navigation characteristics of AUV to select the model for fitting. In the spatial structure detection module and the distance ratio detection module, we used the short-time high-precision characteristics of INS and the error propagation model of INS to perform mismatch detection. Simulation tests are performed on a geomagnetic map with resolution of 0.3′×0.3′. The simulation results show that the proposed method can detect the mismatched points accurately in real time. The matched trajectory gains better reliability after eliminating the mismatched points.

The proposed method can correct the output of DBRN, but it does not improve DBRN itself. The method of performing closed-loop correction on the position error of INS should be discussed in the next step.

## Figures and Tables

**Table 1:** Simulation conditions of the inertial navigation system (INS).

Parameters	Quantity	Unit
Gyro constant drift	0.02	°/hr
Gyro random drift (1σ)	0.02	°/hr
Accelerometer constant bias	100	μg
Accelerometer random bias (1σ)	100	μg
Velocity	7.71	m/s
Acceleration	0	m/s
Initial angle error	0	°
Azimuth angle	60	°
Initial longitude error	0.1	′
Initial latitude error	0.1	′
Simulation time	10	hr

**Table 2:** Parameters of the geomagnetic base map.

Parameters	Quantity	Unit
Number of grid points	840 × 840	points
Grid step	0.3	′
Minimum value	−719.21	nT
Maximum value	253.44	nT
Mean	−2.46	nT

**Table 3:** Configuration of the vector iterated closest contour point (VICCP) algorithm.

Parameters	Parameter Values
Maximum number of iterations	500
Number of sampling points per sequence	13
Sampling interval	5 min
The variance of the magnetic anomaly measurement noise	1 nT

**Table 4:** Statistic results of the RSOC-based mismatch diagnostic algorithm and the proposed method.

Detection Method	*CD*	*TN*	*CR*	*DR*
RSOC-based mismatch diagnostic algorithm	30	34	88.24%	46.88%
Proposed algorithm	45	54	83.33%	70.31%

**Table 5:** Threshold value settings and results.

Threshold Value		*CD*	*TN*	*CR*	*DR*
*T*	ε				
3 grid	0.001	59	86	68.60%	92.19%
3 grid	0.1	41	47	87.23%	64.06%
0.5 grid	0.01	62	97	63.92%	96.88%
7 grid	0.01	36	41	87.90%	56.25%
